# Redox‐Neutral Selenium‐Catalysed Isomerisation of *para*‐Hydroxamic Acids into *para*‐Aminophenols

**DOI:** 10.1002/anie.202100801

**Published:** 2021-03-24

**Authors:** Hsiang‐Yu Chuang, Manuel Schupp, Ricardo Meyrelles, Boris Maryasin, Nuno Maulide

**Affiliations:** ^1^ University of Vienna Institute of Organic Chemistry Währinger Strasse 38 1090 Vienna Austria; ^2^ CeMM—Research Center for Molecular Medicine of the Austrian Academy of Sciences Lazarettgasse 14, AKH BT 25.3 1090 Vienna Austria; ^3^ University of Vienna Institute of Theoretical Chemistry Währinger Straße 17 1090 Vienna Austria

**Keywords:** [2,3]-rearrangement, aminophenol, hydroxamic acid, N−O bond cleavage, selenium

## Abstract

A selenium‐catalysed *para*‐hydroxylation of *N*‐aryl‐hydroxamic acids is reported. Mechanistically, the reaction comprises an N−O bond cleavage and consecutive selenium‐induced [2,3]‐rearrangement to deliver *para*‐hydroxyaniline derivatives. The mechanism is studied through both ^18^O‐crossover experiments as well as quantum chemical calculations. This redox‐neutral transformation provides an unconventional synthetic approach to *para*‐aminophenols.

## Graphical abstract

Prepare for trouble—and make it double: A organoselenium‐catalysed, regiospecific *para*‐hydroxylation starting from *N*‐aryl hydroxamic acids through double [2,3]‐sigmatropic rearrangement to form *para*‐aminophenols was developed. This operationally easy reaction delivers useful *para*‐aminophenol building blocks on small and large scales and its mechanism is supported through quantum chemical calculations as well as labeling experiments.

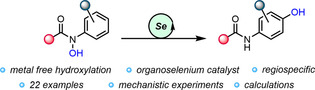

The *para*‐aminophenol motif, epitomized by the century‐old analgesic paracetamol, is an important structural feature in pharmaceuticals and materials. Numerous methods for the preparation of *para*‐aminophenols have been reported ever since Eugen Bamberger discovered the first practical synthesis employing the rearrangement of *N*‐arylhydroxylamine in aqueous sulfuric acid (Scheme 1 a).^[1]^ This process presumably involves the heterolytic cleavage of the N−O bond and subsequent intermolecular addition of water to a nitrenium intermediate. Besides strong Brønsted acids, these N−O bond cleavage/rearrangement events have also been triggered by Lewis acids,^[2]^ thermal activation^[3]^ or transition metals.^[4]^ Pioneering work using Lewis acid‐mediated *ortho*‐migration of a methoxy group was reported by Kikugawa (Scheme 1 b).^[2]^ Later, the same group disclosed the PBu_3_/CCl_4_‐induced *ortho*‐migration of the hydroxyl group in *N*‐acyl‐*N*‐phenylhydroxylamines (Scheme 1 c); minor amounts of the *para*‐isomer were also observed.^[5]^ Ngai described the elegant *ortho*‐trifluoromethoxylation of aniline through a thermal rearrangement process (Scheme 1 d).^[3]^ Recently, Terada reported an in‐depth study of the elegant cobalt‐catalysed [1,3]‐migration of alkoxycarbonyloxyl groups (Scheme 1 e).^[5]^ Interestingly, the large majority of these N−O bond cleavage processes lead to the formation of new C−O bonds with *ortho*‐selectivity. The few approaches achieving *para*‐hydroxylation either require relatively harsh conditions or produce a mixture of *ortho*‐ and *para*‐regioisomers.[^5^, ^6^] To the best of our knowledge, a mild and practical method for regioselective *para*‐hydroxylation still has not emerged.^[7]^

**Scheme 1:**
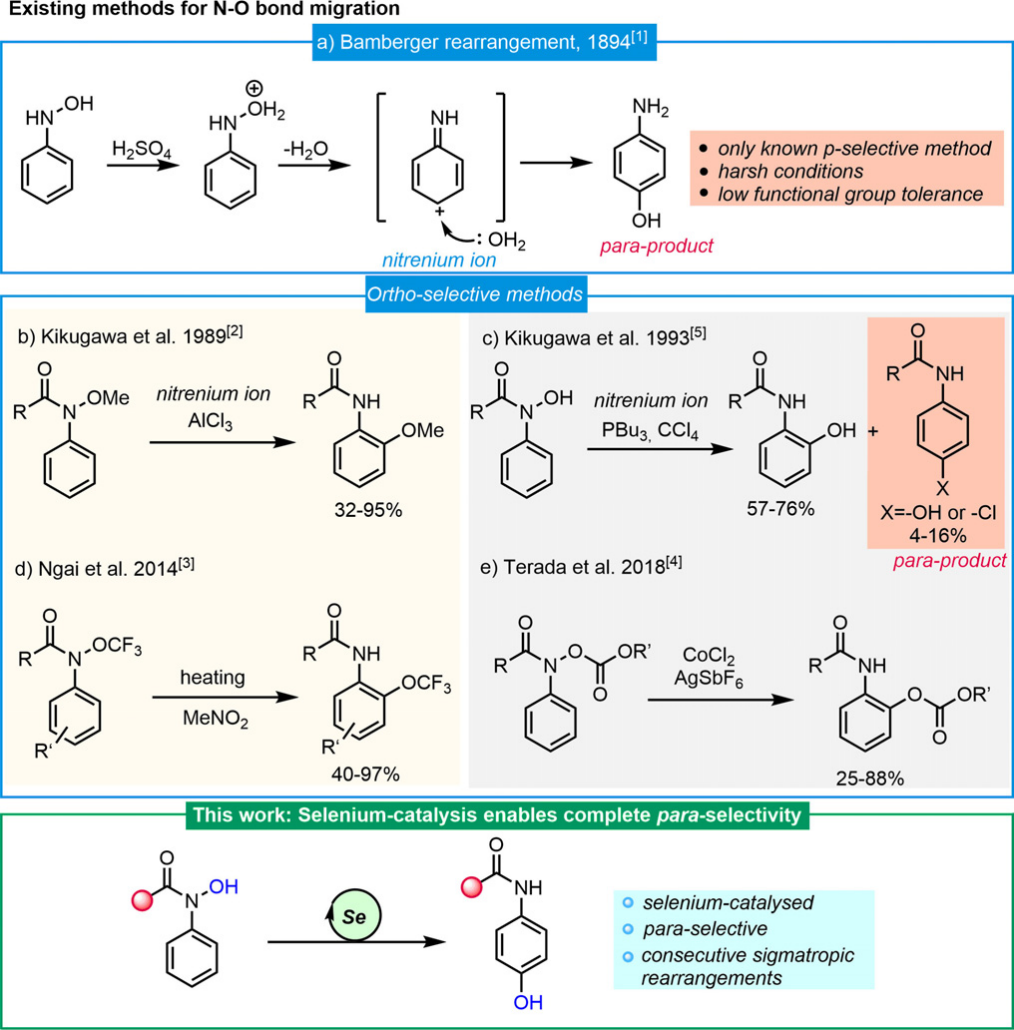
Approaches to N−O bond cleavage/oxygen‐migration reactions and work presented herein.

Selenium is an essential oligoelement, perhaps best known for its occurrence in selenocysteine.[^8^, ^9^, ^10^] Within organic synthesis, organoselenium reagents have also emerged as unique catalysts for oxidation,^[11]^ reduction,^[12]^ C−C/C−X bond formation and rearrangements.[^13^, ^14^, ^15^] The heavier selenium shows distinct properties when compared to the other chalcogens.^[16]^ Herein we present a new selenium‐catalysed, redox‐neutral *para*‐selective hydroxylation starting from hydroxamic acids via consecutive [2,3]‐rearrangements to form *para*‐aminophenols (Scheme 1 f).

In initial efforts, we treated hydroxamic acid **1** with one equivalent of PhSeBr. Gratifyingly, the *para*‐aminophenol **2** was obtained in 72 % isolated yield (Table 1, entry 1). Encouraged by this early result, we realized that reducing the loading of phenylselenyl bromide to 10 mol % still afforded *para*‐aminophenol **2** initially in 66 % yield (entry 2). It is noteworthy that the catalytic process, while requiring increased reaction time to reach full conversion, resulted in only a slight decline in yield. We noted that *para*‐hydroxylation catalysed by PhSeCl gave almost the same yield as with PhSeBr (entry 3). Changing the catalyst to *N*‐(phenylselenyl)‐phthalimide or 2‐nitrophenyl selenocyanate led to 40 % and 35 % yields of *para*‐aminophenol **2**, respectively (entries 4 and 5). To increase the electrophilicity of the selenium reagent, a combination of PhSeCl and AgOTf was employed but gave only 25 % yield of **2** (entry 6). PhSeSePh was ineffective and resulted in recovery of starting material. After these initial observations we elected phenylselenyl bromide as the catalyst for further investigations. In subsequent experiments, several solvents were examined. Dichloromethane, acetonitrile and ethereal solvents were all found to be suitable for this reaction (Table 1, entry 8–12). 1,4‐dioxane was eventually elected as the best system, since its high boiling point allows more flexibility for recalcitrant substrates (vide infra).

**Table 1:** Investigation of selenium catalysts. [a] Reactions were carried out at 0.2 M concentration. [b] Yields were determined by NMR using trimethoxybenzene as internal standard. [c] isolated yield. 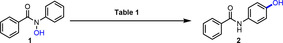

Entry	Reagent	Solvent^[a]^	Temperature	Time	Yield^[b]^
1	PhSeBr (1 equiv)	1,4‐dioxane	rt	1 h	72 %^c^
2	PhSeBr (10 mol %)	1,4‐dioxane	rt	3 h	66 %
3	PhSeCl (10 mol %)	1,4‐dioxane	rt	6 h	67 %
4	*N*‐(Phenylseleno)‐phthalimide (10 mol %)	1,4‐dioxane	rt	12 h	40 %
5	2‐nitrophenyl selenocyanate (10 mol %)	1,4‐dioxane	rt	18 h	35 %
6	PhSeCl (10 mol %) AgOTf (10 mol %)	1,4‐dioxane	rt	18 h	25 %
7	PhSeSePh (10 mol %)	1,4‐dioxane	rt	18 h	0 %
8	PhSeBr (10 mol %)	1,4‐dioxane	rt	3 h	79 % (76 %)^c^
9	PhSeBr (10 mol %)	MeOH	rt	3 h	29 %
10	PhSeBr (10 mol %)	CH_2_Cl_2_	rt	3 h	73 %
11	PhSeBr (10 mol %)	MeCN	rt	3 h	76 %
12	PhSeBr (10 mol %)	THF	rt	3 h	81 %

With suitable reaction conditions in hand, we turned our attention towards the scope of this selenium‐catalysed hydroxylation (Scheme 2). As shown, the transformation tolerates a broad range of functionalities, including the sterically hindered pivalamide **4 a** and adamantylamide **4 b**, as well as the highly strained cyclobutane **4 c**. Notably, higher yields and shorter reaction times are achieved for substrates carrying an electron‐deficient benzamide fragment (see **4 d**, **2**, **4 e**). This appears to correlate with a correspondingly weaker N−O bond in those substrates. Also tolerated are cinnamylamide **4 f** and styrene‐amide **4 g**, albeit with slightly diminished yields. Next, a variety of different substituents at the *N*‐aryl ring were investigated. Naphthalene **3 i** reacted smoothly to give aminophenol **4 i**. Noteworthy, the congested 3,4‐dimethyl‐substituted substrate **3 k** and 2,6‐dimethyl‐substituted substrate **3 l** both led to the corresponding *para*‐aminophenols. Furthermore, our protocol was also applicable towards various halogen‐substituted substrates to afford the desired *para*‐aminophenol (**4 o**–**4 t**). Electronic effects at the *N*‐arene ring significantly affected the reaction yield: while the electron‐rich 4‐methoxyphenyl substrate **3 m** was high‐yielding at room temperature, *N*‐electron deficient hydroxamic acids (**4 n, 4 p, 4 q**) required higher temperature to form the corresponding *para*‐aminophenol in moderate yields.^[17]^

**Scheme 2:**
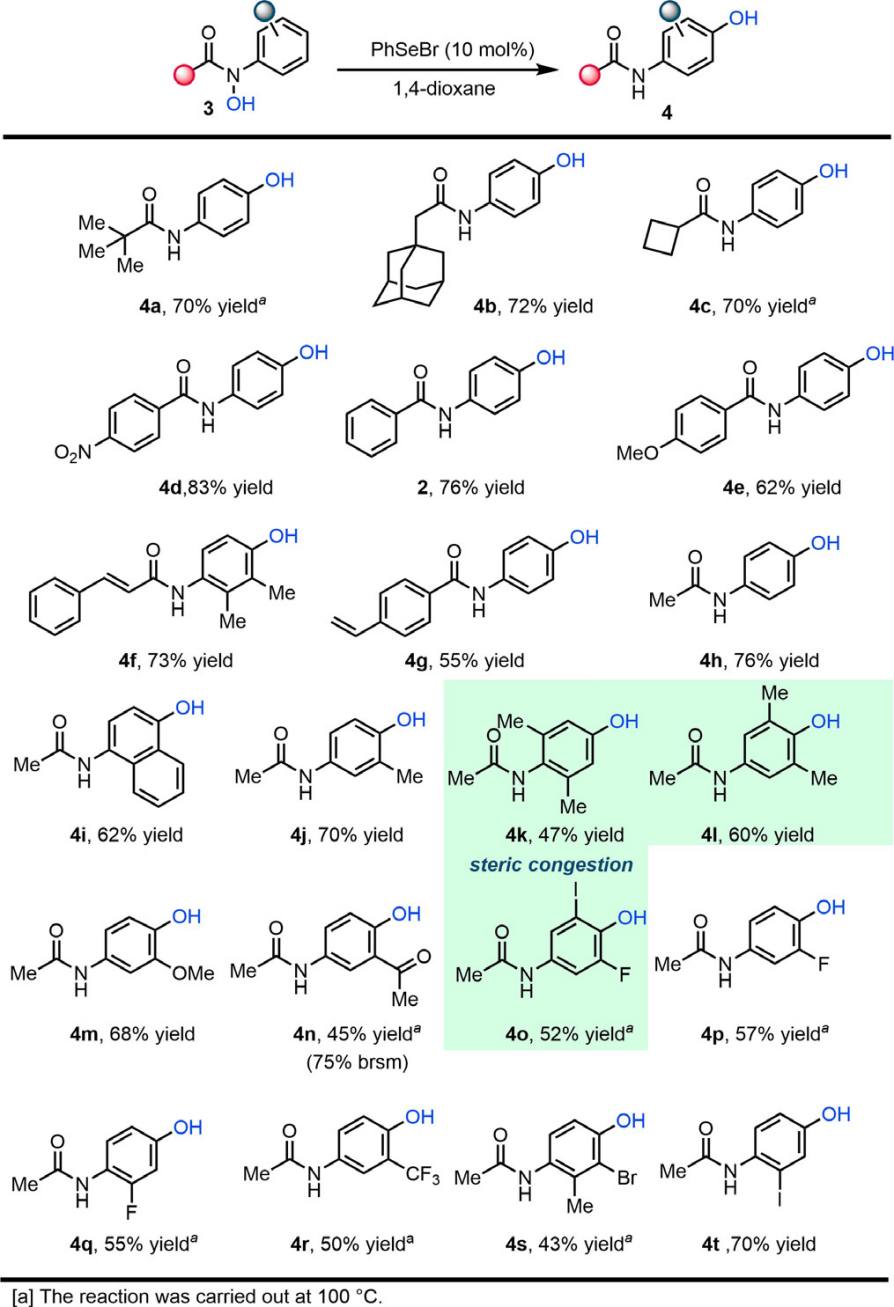
Scope of selenium‐catalysed *para*‐hydroxylation. Yields refer to pure, isolated products.

In order to elucidate the mechanism of the reported reaction, we carried out ^18^O‐labelling studies, as well as quantum chemical calculations (see Supporting Information for additional details). In the event, upon reaction of 3 h* and **1** as a 1:1 mixture under the optimised conditions, no transfer of ^18^O into product **2** was found (Scheme 3). This strongly suggests that the process at hand is an *intramolecular* transformation.

**Scheme 3:**
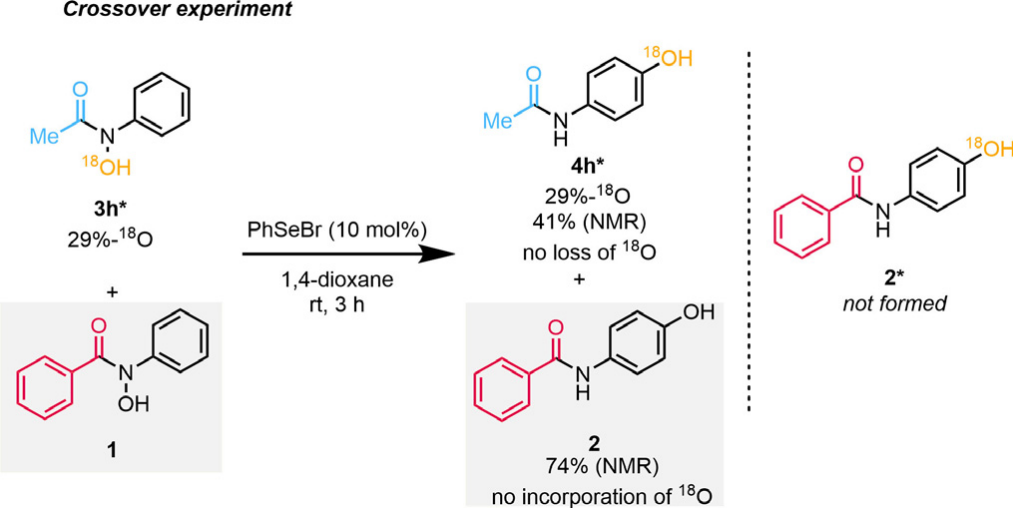
^18^O‐crossover experiment. Yields were determined by NMR using trimethoxybenzene as internal standard.

Quantum chemical calculations have been performed to understand the mechanism of this process. The computed catalytic cycle is presented in Scheme 4 (see Supporting Information for computational details). The active species **A** is obtained following combination of substrate **3 h** with PhSeBr and an internal proton transfer, in line with reported electrophilic selenium reactivity.^[18]^ The first step **A→B** is an exergonic [2,3]‐sigmatropic rearrangement with N−O bond cleavage and *ortho*‐attack of selenium, followed by a barrierless proton transfer **B→C**. Intermediate **C** then undergoes a second proton transfer, preceding the second [2,3]‐sigmatropic rearrangement **D→E**. This step involves concerted Se−C bond cleavage and the formation of a new C−O bond leading to the *para*‐O‐aryl intermediate **E**. The fifth step **E→F** is the highly thermodynamically and kinetically favorable (Δ*G*=−28 kcal mol^−1^, Δ*G* ^*≠*^=7 kcal mol^−1^) re‐aromatization assisted by a second substrate molecule. The last step ultimately closes the catalytic cycle yielding the final product and regenerating intermediate **A**. Interestingly, the apparent activation energy of the cycle, Δ*G* ^*≠*^=25 kcal mol^−1^, is determined by the final step, the intermolecular proton transfer.

**Scheme 4:**
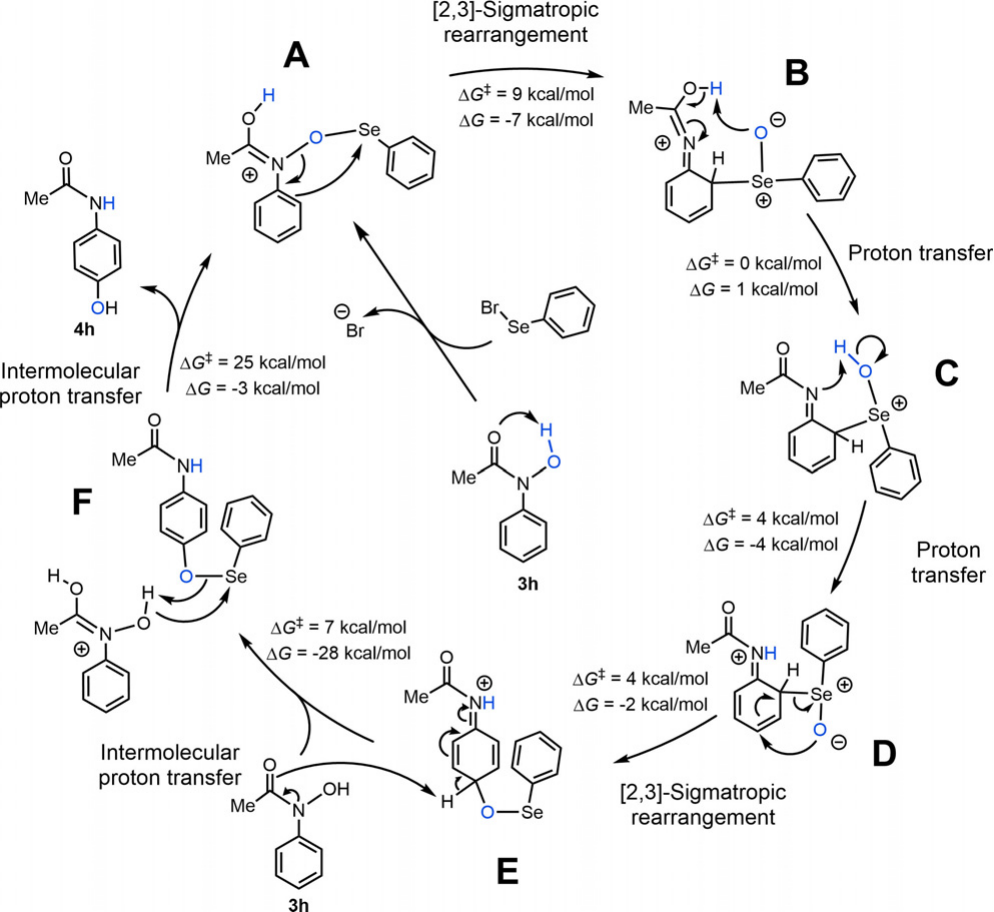
Computed catalytic cycle at the PBE0‐D3BJ‐SMD(THF)/def2‐TZVP//PBE0‐D3BJ‐SMD(THF)/def2‐SVP level of theory. The Gibbs free energies (Δ*G*) and the activation energies (Δ*G* ^*≠*^) are presented for each individual step.

The proposed mechanism highlights the critical role of the substrate itself in the deprotonation of intermediate **E**, in agreement with the base‐free conditions that are employed.

This redox‐neutral, regioselective hydroxylation can be deployed in a number of synthetically relevant contexts (Scheme 5). Practolol (Scheme 5 a, compound **5**) is a known beta‐adrenergic blocking agent, often used for the treatment of cardiovascular diseases, and it has been previously prepared by various routes.[^19^, ^20^] In our gram‐scale approach, hydroxamic acid **3 h** was exposed to selenium‐catalysed *para*‐hydroxylation providing 72 % yield of *para*‐aminophenol **4 h**. Ether synthesis with epichlorhydrin, followed by epoxide opening by isopropylamine gave practotol **5** in 56 % yield over two steps. Next, we targeted diloxanide furoate **8**, a luminal amoebicide widely used as the treatment against amoeba infections (Scheme 5 b).^[21]^ Readily prepared dichloroacetyl hydroxamic acid **6** was subjected to selenium‐catalysis to yield the corresponding *para*‐dichloroacetyl aminophenol **7** in 57 % yield. Introduction of the furoyl group and methylation completed the synthesis of **8**.

**Scheme 5:**
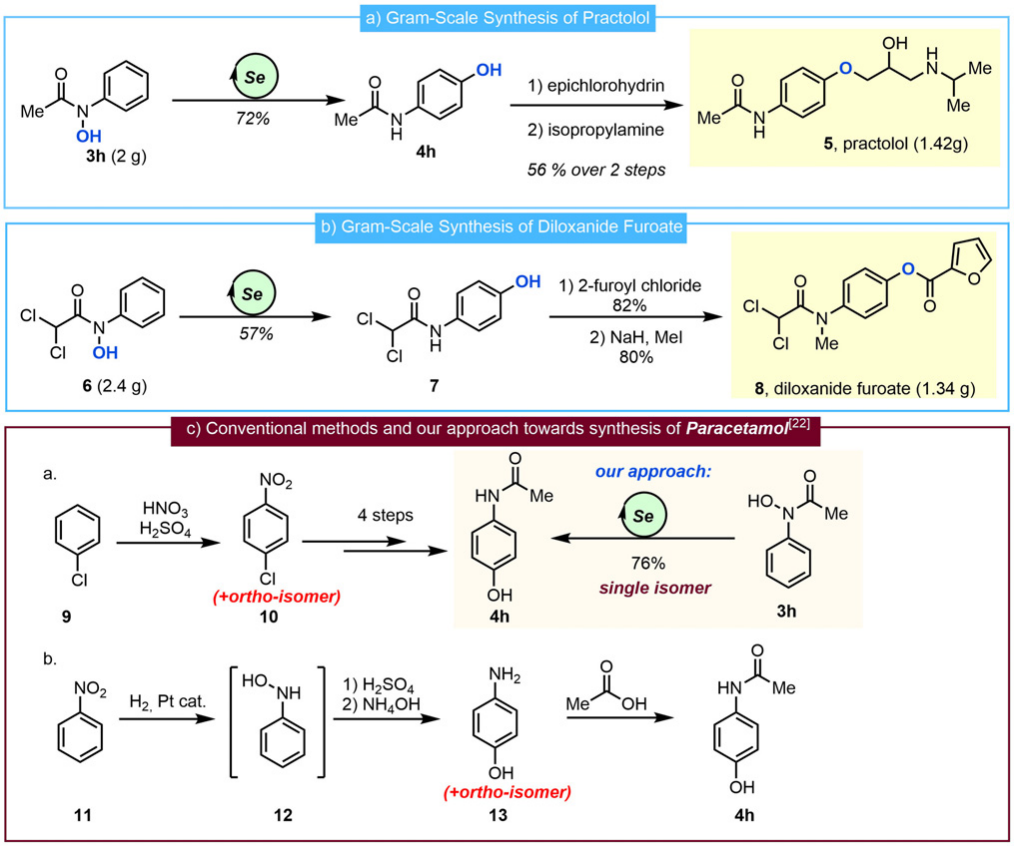
Gram‐scale syntheses of a) practolol and b) diloxanide furoate using Selenium‐catalysis. c) Comparison of methods for the preparation of paracetamol and our one‐step, regioselective approach.

Finally, Paracetamol/*para*‐acetaminophenol **4 h**, one of the most commonly used and produced drugs worldwide, is conventionally prepared by a few different methods. Representative approaches are depicted in Scheme 5 c.^[22]^ The first route involves a nitration of chlorobenzene **9** that also produces *ortho*‐chloronitrobenzene as side product.^[22]^ Processes using a Bamberger reaction also form significant amounts of *ortho*‐aminophenol.^[22]^ In contrast, our selenium‐catalysed *para*‐hydroxylation offers a highly regioselective, alternative solution as it generates *para*‐aminophenol **4 h** from simple precursor **3 h** as the single regioisomer in excellent yield.

In conclusion, we have reported a catalytic method for the synthesis of *para*‐aminophenols from the corresponding arylhydroxamic acids. The catalytic reaction proceeds via a unique electrophilic selenium‐induced N−O bond cleavage event followed by a successive [2,3]‐rearrangement to form the *para*‐aminophenol assisted by another substrate molecule. The mechanism is supported by ^18^O‐crossover experiments as well as quantum chemical calculations. This operationally easy process tolerates a broad range of functional groups and can easily be applied, for example, to prepare practotol **5** and diloxanide furoate **8** in gram‐scale.

## Conflict of interest

The authors declare no conflict of interest.

## Supporting information

As a service to our authors and readers, this journal provides supporting information supplied by the authors. Such materials are peer reviewed and may be re‐organized for online delivery, but are not copy‐edited or typeset. Technical support issues arising from supporting information (other than missing files) should be addressed to the authors.

Supplementary

Supplementary

Supplementary
